# Chitinase 3 like 1 suppresses the stability and activity of p53 to promote lung tumorigenesis

**DOI:** 10.1186/s12964-019-0503-7

**Published:** 2020-03-04

**Authors:** Kyung-Ran Park, Hyung-Mun Yun, Kyeongwon Yoo, Young Wan Ham, Sang Bae Han, Jin Tae Hong

**Affiliations:** 1 Department of Oral and Maxillofacial Pathology, School of Dentistry, Kyung Hee University, Seoul, 02453 Republic of Korea; 2 KRIBB/Bio-venture Center 113 125 Gwahak-ro, Yuseong-gu, Daejeon 34141 Republic of Korea; 3 Department of Chemistry and Biochemistry, Brigham Young University, Provo, UT USA; 4 College of Pharmacy and Medical Research Center, Chungbuk National University, Osongsaengmyeong 1-ro 194-21, Osong-eup, Heungduk-gu, Cheongju, Chungbuk 361-951 Republic of Korea

**Keywords:** Intracellular Chi3L1, p53, Knockout mice, Lung cancer, Tumor development

## Abstract

**Background:**

Chitinase 3 like 1 protein (Chi3L1) is expressed in several cancers, and a few evidences suggest that the secreted Chi3L1 contributes to tumor development. However, the molecular mechanisms of intracellular Chi3L1 are unknown in the lung tumor development. **Methods:** In the present study, we generated Chi3L1 knockout mice (Chi3L1^KO(−/−)^) using CRISPR/Cas9 system to investigate the role of Chi3L1 on lung tumorigenesis.

**Results:**

We established lung metastasis induced by i.v. injections of B16F10 in Chi3L1^KO(−/−)^. The lung tumor nodules were significantly reduced in Chi3L1^KO(−/−)^ and protein levels of p53, p21, BAX, and cleaved-caspase 3 were significantly increased in Chi3L1^KO(−/−)^, while protein levels of cyclin E1, CDK2, and phsphorylation of STAT3 were decreased in Chi3L1^KO(−/−)^. Allograft mice inoculated with B16F10 also suppressed tumor growth and increased p53 and its target proteins including p21 and BAX. In addition, knockdown of Chi3L1 in lung cancer cells inhibited lung cancer cell growth and upregulated p53 expression with p21 and BAX, and a decrease in phosphorylation of STAT3. Furthermore, we found that intracellular Chi3L1 physically interacted and colocalized with p53 to inhibit its protein stability and transcriptional activity for target genes related with cell cycle arrest and apoptosis. In lung tumor patient, we clinically found that Chi3L1 expression was upregulated with a decrease in p53 expression, as well as we validated that intracellular Chi3L1 was colocalized, reversely expressed, and physically interacted with p53, which results in suppression of the expression and function of p53 in lung tumor patient.

**Conclusions:**

Our studies suggest that intracellular Chi3L1 plays a critical role in the lung tumorigenesis by regulating its novel target protein, p53 in both an in vitro and in vivo system.

## Background

Lung cancer is the leading cause of cancer related mortality worldwide [1], and approximately 10–15% are caused by genetics [2]. Most (approximately 85%) of lung cancers are of the non-small-cell type (NSCLC) [3]. Major clinical breakthroughs in late stage lung cancer have been facilitated by the recent advent of patient selection based upon tumor genetic profiles that have promoted a personalized medicine approach for non-small cell lung patients (NSCLC) [4]. Typical treatment for NSCLC is chemotherapy for inhibiting several receptors and intracellular signaling such as epidermal growth factor receptor (EGFR), vascular endothelial growth factor receptor (VEGFR), insulin-like growth factor 1 receptor (IGF-1R), RAS proto-oncogene (RAS), mammalian target of rapamycin (mTOR), and signal transducer and activator of transcription 3 (STAT3) [5–8]. However, the targeted chemotherapies are their effects only in a small fraction of lung cancer patients who have the corresponding molecular alteration that responds to the appropriate treatment [3, 5]. In addition, most of the chemotherapeutic agents have been shown to cause treatment related side effects [3, 9, 10]. Thus, development of new treatment targets still needs to increase effective targeted therapies without side effects and potential benefit for more lung cancer patients.

Chitinase 3–like-1 (Chi3L1), also called a breast regression protein 39 (BRP-39) in mouse and YKL-40 in human, is known as a secreted glycoprotein and prototypic mammalian chitinase like protein [11, 12]. Increased expression of Chi3L1 protein and mRNA have been shown in various disease models and states including rheumatoid arthritis schizophrenics, inflammatory bowel disease, chronic obstructive pulmonary disease, asthma, diabetes, and atherosclerosis [13–19], Especially, Chi3L1 expression has been found in a variety of cancer cells such as breast, lung, prostate, colon, rectum, ovary, kidney, breast, glioblastomas, and malignant melanoma [20–26].

Our previous published data showed that spontaneous lung tumor incidence was lower in the presenilin mutant mice and the proteomic analysis showed that the presenilin mutant mice had very lower levels of chitinase like protein level in the lung tissue [27]. A paper shows Chi3L1 negatively regulates Type 1 T helper (Th1) and Cytotoxic T lymphocyte (CTL) functions on lung metastasis [22]. Chi3L1 play a critical role as a Type 2 T helper (Th2) promoting cytokine that is present at high levels in the tumor microenvironment and in the serum of cancer patients [28, 29]. It was also reported that Chi3L1 is increased in serum and lung of patients suffering from idiopathic pulmonary fibrosis [30], and high serum Chi3L1 level in patients with small cell lung cancer is related to early death [31]. These results indicate that Chi3L1 could be significant for lung tumor development.

Interestingly, it was reported that Chi3L1 was known as a secreted protein in various cell-types [11, 12], but Chi3L1 was also localized within the cytoplasm and nucleus of monocyte derived dendritic cells [32]. To date, there are no studies for the direct role of intracellular Chi3L1 on lung tumorigenesis, although higher expression and level of Chi3L1 in several cancer cells and tumor patient tissues have been reported. Considering that high serum Chi3L1 is a poor prognostic marker in lung cancer patients [33], it should be investigated for intracellular Chi3L1 expression and direct roles and mechanisms on lung tumorigenesis by intracellular Chi3L1.

In the present study, we demonstrated the critical role of intracellular Chi3L1 in both an in vitro and in vivo system using Chi3L1 knockout mice (Chi3L1^KO(−/−)^) generated by CRISPR/Cas9 system, lung cancer cells, and lung tumor patient samples. Our data indicate intracellular Chi3L1 as a key regulatory protein in lung tumorigenesis.

## Methods

### Human samples

Human lung cancer and normal lung tissues from 15 lung cancer patients were obtained from Keimyung University Dongsan Medical Center, Chonnam National University Hospital, and Chonbuk National University Hospital. All studies for human samples were conducted in accordance with the Declaration of Helsinki and were approved by the Ethics Committee of Chungbuk National University Medical Centre (IRB No.: CBNU-IRB-2011-U01).

### Animals

Mice were housed in standard cages in an Assessment and Accreditation of Laboratory Animal Care credited specific pathogen-free (SPF) animal facility on a 12 h light-12 h dark cycle. All protocols involving mice in this study were reviewed and approved by the Chungbuk National University Institutional Animal Care and Use Committee (IACUC) and complied with the Korean National Institute of Health Guide for the Care and Use of Laboratory Animals (CBNUA-792-15-01).

### DNA constructs for the CRISPR RNAs

Single guide RNAs (sgRNAs) targeting a site of genome corresponding N-terminal region of chi3l1 were designed using ZiFiT (http://zifit.partners.org/ZiFiT/) program. The candidates of target nucleotides having a potential off-target with 1 or 2 base mismatch were avoided for the design. The two complimentary oligos of each sgRNA were annealed and cloned in pT7-gRNA vector which is a vector designed for synthesis of sgRNA [34].

### In vitro sgRNA synthesis and purification

In vitro transcription of sgRNAs and RNA purification were performed using MEGAshortscript T7 kit (Ambion) according to the manufacturer’s instructions.

### Microinjection of one-cell zygotic embryos

Microinjection was performed in the fertilized eggs from C57BL/6 mice. The embryos were harvested in M2 medium and cultured in M16 medium for 2–3 h. The mixture of sgRNA (100 ng/μl) and Cas9 protein (80 ng/μl) was injected into the cytoplasm of the one-cell stage embryos. Injected embryos were overnight incubated in the culture media prior to embryo transfer into pseudo-pregnant female mice [35].

### Genotyping by T7E1 assay and sequencing analysis

Genomic DNAs were extracted from toes or tails of the progenies and subjected to PCR. PCR amplicons were denatured and slowly reannealed to facilitate heteroduplex formation. The reannealing procedure consisted of a 5-min denaturing step at 95 °C, followed by cooling to 85 °C at − 2 °C per second and further to 25 °C at − 0.1 °C per second. Reannealed amplicons were treated with 5 units of T7 endonuclease I (New England BioLabs) for 30 min at 37 °C then analyzed by agarose gel electrophoresis. To check the potential off-target effects, the genomic regions encompassing the potential off-target sites with 1 or 2 base-mismatch were PCR amplified and subjected to T7E1 assay or sequencing analysis. For PCR genotyping assay, primer sets amplifying region of exon 3 were designed as followed. Forward: 5-GAGTTTAGTATCCCATATCACC-3, reverse: 5-GGCCACATATTTTGTCACTCAT-3, which amplify a PCR fragment of 600 base pairs long.

### Lung metastasis model

B16F10 cells were injected into the lateral tail veins of Chi3L1^KO(−/−)^ mice and wild type mice (1 × 10^4^ cells in /100 μl phosphate-buffered saline (PBS) per animal). 8 weeks after the injections, animals were sacrificed and the tumor lung metastases were counted on the lung surface. Metastases were counted in all lobes of the lung, except for the middle lobe where the primary tumors localized. Data are presented as the number of tumor nodules per lung.

### Allograft animal model

B16F10 cells were implanted subcutaneously (s.c.) (1 × 10^6^ tumor cells/0.1 ml PBS/animal) into upper dorsal region of the mice with a 27 gauge needle. Implantation tumors visually detected in injected region of the mice after 7 days. The weight and tumor volume of the animals were monitored twice per week during 27 days. The tumor volumes were measured with vernier calipers and calculated using the following formula: A × B^2^ / 2, where A is the larger and B is the smaller of the two dimensions. At the end of the experiment, the animals were sacrificed and the tumors were separated from the surrounding muscles and dermis, excised and weighed.

### Immunohistochemistry

Tumor tissues were fixed in formalin and embedded in paraffin for examination. Sections were stained with hematoxylin and eosin (H&E) and analyzed by immunohistochemistry. The sections were blocked for 30 min with 5% BSA diluted in 1X PBS and incubated with specific primary antibodies (1:200 dilution). The next day, immunological detection was started with incubation in horseradish peroxidase (HRP)-conjugated secondary antibodies (1:500, Jackson ImmunoResearch) for 1 h at room temperature. After washing with 1X PBS, chromogen development was performed with 0.02% 3,3′-diaminobenzidine tetrahydrochloride (DAB, Vector Laboratories, Burlingame, CA). Finally, the sections were dehydrated with ethanol, cleared with xylene, and mounted with Permount (Fisher Scientific, Rockford, IL), and evaluated on a light microscopy (ZIESS, Oberkochen, Germany).

### Western blot analysis

Western blot analysis was done as described previously [36]. Cells were washed twice with ice-cold PBS, and lysed in 20 mM Tris-HCl buffer (pH 7.4) containing a protease inhibitor mixture (0.1 mM PMSF, 5 mg/mL aprotinin, 5 mg/mL pepstatin A, and 1 mg/mL chymostatin). Protein concentration was determined using Bradford reagent (Bio-Rad, Hercules, CA). Equal amounts of lysate (20 μg) were resolved by sodium dodecyl-polyacrylamide gel electrophoresis (SDS-PAGE), and subsequently transferred to a polyvinylidene fluoride (PVDF) membrane (Millipore, Bedford, MA). Thereafter, the membrane was blocked with 1 × TBS containing 0.05% Tween 20 (TBST) and 5% skim milk or 2% BSA for 1 h at room temperature. After blocking, the membranes were incubated overnight at 4 °C with the respective primary antibodies, washed with 1 × TBST, and then incubated with diluted horseradish peroxidase (HRP)-conjugated secondary antibodies (1:10,000, Jackson ImmunoResearch, West Grove, PA) for 1 h at room temperature. After three washes, the bound antibodies were detected using an enhanced chemiluminescence (ECL) kit (Millipore, Bedford, MA). The intensity of the bands was measured using the Fusion FX 7 image acquisition system (Vilber Lourmat, Eberhardzell, Germany).

### Adenoviral vector generation for ad-shChi3L1

Ad-shChi3L1 was constructed by Sirion Biotech (Martinsried, Germany). The U6-shRNA-SV40-pA region of the pO6A5 shuttle vector was transferred via recombination in a bacterial artificial chromosome vector, containing the genome of the replication Ad5-based vector deleted for the E1/E3 genes (involved in replication and immunomodulation, respectively). Adenoviral particles were produced by construction of the shRNA expression shuttle vector into HEK-293 cells. Cloning success for the resultant vector was verified by restriction analysis and DNA sequencing.

### Cell culture, transfection, and transduction

A549 human lung cancer cells and and B16F10 mouse skin melanoma were obtained from the American Type Culture Collection (Manassas, VA, USA). RPMI1640, penicillin, streptomycin, and fetal bovine serum were purchased from Invitrogen (Carlsbad, CA, USA). A549 cells were grown in RPMI1640 with 10% fetal bovine serum, 100 U/ml penicillin, and 100 μg/ml streptomycin at 37 °C in 5% CO_2_ humidified air. B16F10 cells were grown in DMEM with 10% FBS, 100 U/mL penicillin, and 100 μg/mL streptomycin, at 37 °C in 5% CO_2_ humidified air. For transfection and transduction, we used Lipofectamine 3000, Lipofectamine RNAiMAX (Invitrogen), and Ad-shChi3L1.

### Cell proliferation assay

Cell proliferation was measured by an 3-[4,5-dimethylthiazol-2-yl]-2,5-diphenyltetrazolium bromide (MTT) assay to detect NADH-dependent dehydrogenase activity as previously described [37]. 5 mg/ml 3-(4,5-dimethylthiazol-2-yl)-2,5-diphenyltetrazolium bromide (MTT; Sigma-Aldrich, St. Louis, MO) diluted in PBS was added to the cells, which was then incubated for 2 h to allow MTT to metabolize to formazan. Absorbance was measured at 570 nm (Beckman Coulter, Fullerton, CA). The data were normalized to their respective controls and are presented as a bar graph.

### Ubiquitination assay

A549 cells were transfected with control siRNA or Chi3L1 siRNA using Lipofectamine RNAiMAX (Invitrogen). After transfection, the soluble lysates were incubated with anti-p53 antibody (Santa Cruz Biotechnology, Santa Cruz, CA) at 4 °C and incubated with Protein A agarose (Sigma-Aldrich), and then washed six times. Immunoprecipitates were eluted by boiling for 10 min at 95 °C in SDS sample buffer followed by Western blotting with anti-ubiquitin (1:1000, Santa Cruz Biotechnology) or anti-p53 (1:1000, Cell Signaling Technology, Beverly, MA) antibodies.

### Co-immunoprecipitation

Cells and tissues were gently lysed with lysis buffer for 1 h on ice and then centrifuged at 15,000 g and 4 °C for 15 min, and the supernatant was collected. After the lysates were precleared with 50 μl of Protein A agarose (Sigma-Aldrich) for 2 h, the precleared lysates were incubated with 2 μg of each specific antibody overnight at 4 °C and then incubated with 50 μl of Protein A agarose for 4 h at 4 °C and were washed seven times. Immune complexes were eluted by boiling for 10 min at 95 °C in SDS sample buffer, followed by Western blotting with mouse primary anti-Myc (1:1000, Cell Signaling Technology) or mouse primary anti-p53 (1:1000, Santa Cruz Biotechnology) antibodies for cells, and rabbit primary anti-Chi3L1 (1:1000, abcam, Cambridge, MA) or mouse primary anti-p53 (1:1000, Santa Cruz Biotechnology) antibodies for tissues.

### Molecular docking model

The docking of Parkin with p53 was examined using the rigid-body docking program ZDOCK 3.0.2 on the ZDOCK server (http://zdock.umassmed.edu). p53 (PDB ID: 1TUP monomer) and Chi3L1 (PDB ID: 1NWR monomer) without DNA were used for docking. Docking experiments were carried out without selecting or blocking residues.

### p53-transcriptional activity

The pGL4.38[luc2P/p53 RE/Hygro] Vector contains two copies of a p53 response element (p53 RE) that drives transcription of the luciferase reporter gene luc2P (*Photinus pyralis* was purchased from Promega (Madison, WI). 24 h after transfection into A549 lung cancer cells, luciferase activity was measured using the luciferase reporter assay system (Promega) according to the manufacturer’s protocol.

### Immunofluorescence

Cells and lung cancer patient tissue array with normal colon tissues as control (US Biomax Inc., Rockville, MD) were blocked with 3% BSA diluted in PBS for 1 h and incubated with primary antibodies for overnight at 4 °C. Subsequently, the cells and tissue sections were incubated with an anti-mouse secondary antibody labeled with Alexa-Fluor 488 (1:500 dilution, Invitrogen) and anti-rabbit secondary antibody labeled with Alexa-Fluor 568 (1:400 dilution, Invitrogen) for 2 h at room temperature. Next, the cells and tissue sections were incubated with 4′,6-diamidino-2-phenylindole (DAPI) for 15 min at 37 °C. Finally, the cells and sections were rinsed, mounted, and viewed on on a confocal LSM 510 Laser Scanning microscope (Zeiss, Gottingen, Germany). A negative control was included in all experiments by omitting the primary antibody.

### Data analysis

The data were analyzed using the GraphPad Prism 5 software ((GraphPad Software, Inc., San Diego, CA). Data are presented as mean ± SEM. Statistical significance was evaluated by one-way analysis of variance (ANOVA) and the differences were assessed by the Dunnett’s test. A value of *P* < 0.05 was considered to indicate statistical significance.

## Results

### Chi3L1 accelerates lung tumorigenesis in metastasis and allograft model in vivo

**Fig. 1:**
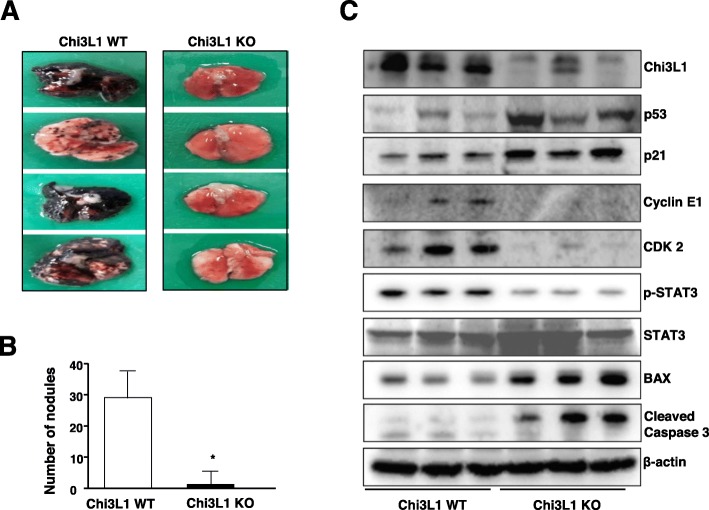
Chi3L1^KO(−/−)^ mice in lung metastasis In Vivo. **a**, **b** B16F10 cells were injected by a single i.v. injection (1 × 10^4^ cells) to induce lung metastasis. At the time of sacrifice, lungs were lavaged (**a**) and used for surface tumor number and diameter measurements (**b**). The results are expressed as mean ± ± SEM. *, *p* < 0.05 compared to wild type mice. **c** Tissue extracts were analyzed by Western blotting. Samples were resolved on SDS-PAGE, and detected with antibodies against Chi3L1, p53, p21, Cyclin E1, phoshp-STAT3, STAT3, Cdk2, BAX, Caspase3, and β-actin. Data shown represent mean ± SEM

To investigate whether Chi3L1 knockout (Chi3L1^KO(−/−)^) mice contribute to lung tumorigenesis, the mice was administrated i.v. with B16F10 melanoma to induce lung metastasis. 3 weeks after the injections, the number of surface lung metastases was significantly lower in Chi3L1^KO(−/−)^ mice than those observed in wild type mice (Fig. 1a and b). As shown in Fig. 1**c**, Western blot analysis showed that p53 and p21 were markedly higher in the lung of Chi3L1^KO(−/−)^ mice, while Cyclin E1, CDK2, and phoshop-STAT3 were significantly lower in the lung of Chi3L1^KO(−/−)^ mice than those in wild type mice We also found that the apoptosis-related marker protein, BAX and cleaved caspase 3 were significantly increased in the meta nodules of Chi3L1^KO(−/−)^ mice, suggesting Chi3L1^KO(−/−)^ mice may reduce lung metastasis by regulating tumor suppressor proteins, cell cycle arrest, apoptosis, and proliferation.

**Fig. 2:**
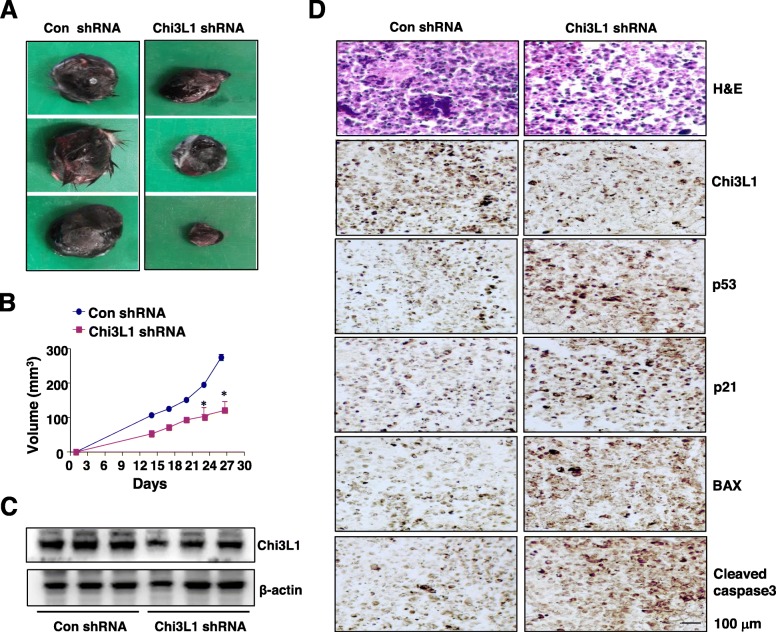
Regulation of Chi3L1 on tumor development in allograft mice. **a-c** Mice inoculated with B16F10 transduced by Chi3L1 shRNA were sacrificed after 27 days. Tumor images (**a**) and tumor volume (**b**) were measured, and tissue extracts were analyzed by Western blotting (**c**). **d** Tumor sections were analyzed by immunohistochemistry for detection of Chi3L1, p53, p21, BAX, and cleaved caspase3 in tumor tissues. Data shown represent mean ± SEM. *, *p* < 0.05 compared to con shRNA

To elucidate the direct function of Chi3L1 in lung tumor growth in vivo, we next investigated lung tumorigenesis in allograft mice inoculated with B16F10 melanoma transduced by a Chi3L1 short hairpin RNA (shRNA)-expressing adenoviral vector (Chi3L1 shRNA mice). Tumor growth was monitored for 27 days after implanted with B16F10 melanoma into the mice. Tumor volume was measured weekly twice, and all mice were killed at the end of the experiment when tumors were dissected and weighted. There was a significant difference in tumor growth between con shRNA and Chi3L1 shRNA mice (Fig. 2a). The tumor volumes and weights of B16F10 lung melanoma transduced by Chi3L1 shRNA were significantly smaller than in those of the control shRNA mice (Fig. 2b). The expression of Chi3L1 in control shRNA and Chi3L1 shRNA mice was confirmed by Western blot analysis (Fig. 2**c**). The histological findings after haematoxylin and eosin (H&E) staining indicated that the tumors in Chi3L1 shRNA mice were significant reduced than those from control shRNA mice **(**Fig. 2d). Immunohistochemical analysis also showed that expression of Chi3L1 was lower in the tumor section of Chi3L1 shRNA mice, whereas the expression of p53 was markedly higher and its target proteins such as p21, BAX, and cleaved cas-3 consequently were increased in the section of Chi3L1 shRNA mice compared to those of control shRNA mice (Fig. 2d).

### Chi3L1 downregulates p53 and its target proteins, and induce the ubiquitination of p53 in the lung cancer cells

**Fig. 3:**
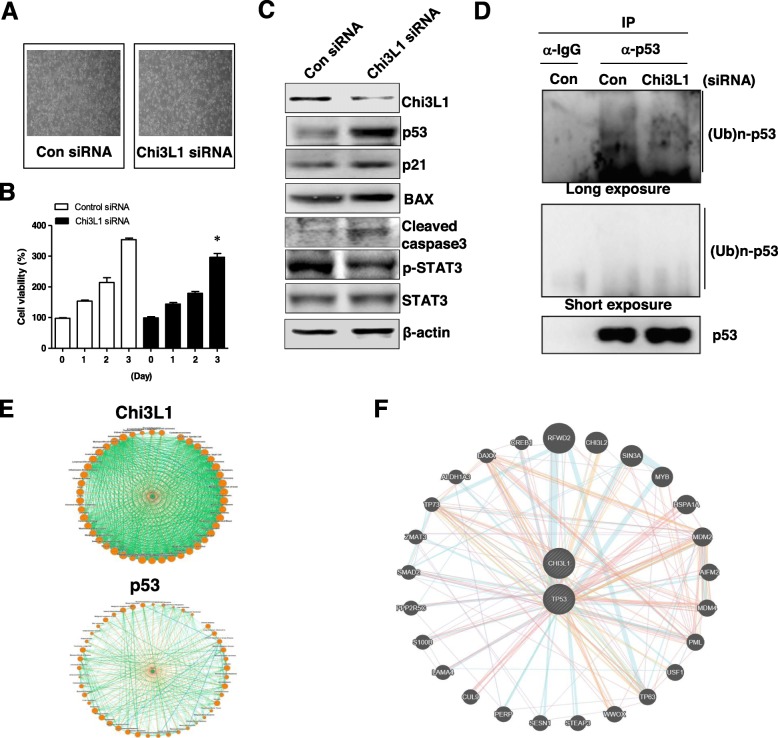
Regulation of Chi3L1 on lung cancer cell growth and tumor suppressor proteins, and diseases and signaling network associated with Chi3L1 and p53. **a**, **b** After A549 lung cancer cells were transfected with Chi3L1 siRNA for 24 h, the morphological changes were observed, and then cell viability was analyzed by MTT assay (**b**). **c** Chi3L1, p53, p21, BAX, cleaved caspase 3, phoshp-STAT3, STAT3, and β-actin were analyzed by Western blotting. **d** After the lung cancer cells were transfected with Chi3L1 siRNA for 24 h, cell lysates were immunoprecipitated with anti-control IgG or anti-p53, and then the cell lysates (*bottom*) and immunoprecipitants (*top*) were then analyzed by immunoblotting with anti-ubiquitin and anti-p53 antibodies. **e** The disease connections of Chi3L1 (*Top*) and p53 (*Bottom*) were analyzed by publicly available Disease-Connect from the mechanism-based disease-disease connections (http://disease-connect.org). **f** The signaling network among Chi3L1, p53, and related signals and was predicted using GeneMANIA (https://genemania.org/). Data shown represent mean ± SEM. *, *p* < 0.05 compared to con siRNA

To verify whether Chi3L1 inhibits lung cancer cell growth, Chi3L1 was knockdowned using Chi3L1 siRNA in A549 lung cancer cells. We found that the lung cancer cell growth was decreased by knockdown of Chi3L1 (Fig. 3a and b. and Additional file 1**:** Figure S1A). Similar to tumor tissues, the expression of p53 and its target proteins (p21 and BAX), and cleaved caspase 3 were increased, whereas the phosphorylation of STAT3 was decreased in the Chi3L1 knockdown lung cancer cells than those of the lung cancer cells transfected with control siRNA (Fig. 3c and Additional file 1: Figure S2A-F). Next, we examined whether knockdown of Chi3L1 affects the ubiquitination of p53 that leads to the degradation of p53. As shown in Fig. 3d, the knockdown of Chi3L1 resulted in a decrease in ubiquitinated high-molecular-weight p53 bands (Fig. 3d**,** *third lane*) in immunoprecipitates using p53 antibody, whereas no signal was detected in immunoprecipitates using control IgG antibody (Fig. 3d, *first lane*).

To predict network connections between Chi3L1 and p53, we searched the diseases and signaling network associated with Chi3L1 and p53 using the mechanism-based disease-disease connections (http://disease-connect.org) (Fig. 3e) and the GeneMANIA in source organism *Homo sapiens* as additional parameters (https://genemania.org/) (Fig. 3f). The network data show that Chi3L1 is also related to the p53 in a variety of diseases and singaling proteins (Fig. 3e and f).

### Chi3L1 localizes in cytoplasm and nucleus, and the intracellular Chi3L1 physically interacts with p53 in the lung cancer cells

**Fig. 4:**
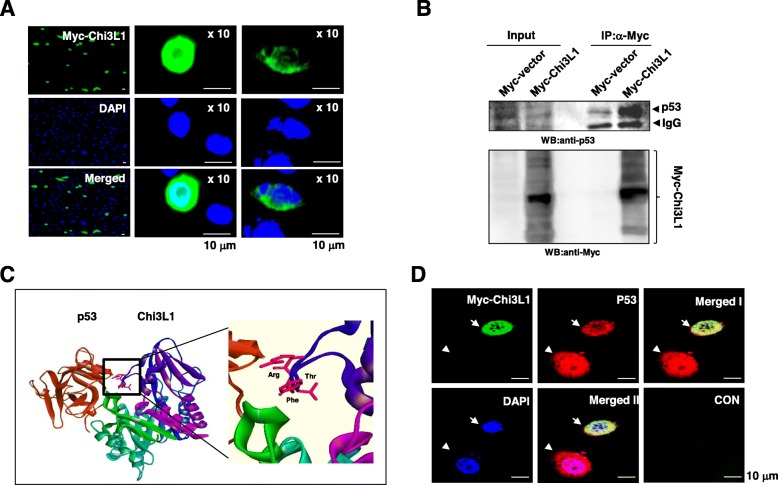
Intracellular co-localization and physical interaction between Chi3L1 and p53 (**a**) After lung cancer cells were transfected with Myc-Chi3L1 for 24 h, the cells were fixed and permeabilized. Myc-Chi3L1 (*green*) was immunostained with mouse anti-Myc antibody, followed by Alex488-conjugated secondary antibodies. And then sections were stained with DAPI (*blue*). **b** The lung cancer cells were lysed and immunoprecipitated with anti-Myc antibody. Cell lysates (*Input*) and immunoprecipitants (*IP*) were then analyzed by Western blotting with anti-Myc and anti-p53 antibodies. **c** Molecular surface representation in the docking model of p53 with Chi3L1. **d** After the lung cancer cells were permeabilized, p53 (*red*) was immunostained with rabbit anti-p53 followed by Alex568-conjugated secondary antibodies and myc-Chi3L1 (*green*) was immunostained with mouse anti-Myc antibody, followed by Alex488-conjugated secondary antibodies. And then sections were stained with DAPI (*blue*). Merged I shows the merged images of Myc-Chi3L1 and p53, and Merged II shows the merged images of myc-Chi3L1, p53, and DAPI. Negative control experiments (CON) were processed with only Alex568- and Alex488-conjugated IgG antibodies. Arrow: Myc-Chi3L1-expressing cell, Arrow head: Myc-Chi3L1-non-expressing cell

We determined the intracellular localization of Chi3L1 in the lung cancer cells. Immunofluorescence data exhibited that Myc-tagged full-length Chi3L1 (myc-Chi3L1) was present in the cytoplasm and nucleus of the lung cancer cells (Fig. 4a). The interaction between Chi3L1 and p53 was next examined using a co-immunoprecipitation (IP) assay. After myc-Chi3L1 was transfected into the lung cancer cells, cell lysates were prepared, immunoprecipitated with anti-Myc antibodies, and subsequently immunoblotted with anti-p53 antibodies. As shown in Fig. 4b, Input data shows the expression of p53 was significantly decreased in the myc-Chi3L1 expressing cells, as well as IP data show that the myc-Chi3L1 bound to endogenous p53 compared to those of transfected cells with control myc-vector. Molecular docking model also indicated possible interactions in the side chain of Arg 86, Phe 87, and Thr 88 of Chi3L1 (Fig. 4c). Although the interaction between two proteins was validated in the lung cancer cells, the association of two proteins in intracellular regions was still undetermined since a common requirement for specific functional interaction between Chi3L1 and p53 is that they must be positioned in same regions. We, therefore, examined whether both proteins are co-localized in the same cellular compartment in the lung cancer cells. Immunofluorescence analysis using confocal microscopy revealed that Chi3L1 was positioned in close proximity for functional interaction between Chi3L1 and p53, which also demonstrated a decrease in the expression of p53 in myc-Chi3L1 expressing cells compared to that of myc-Chi3L1-non-expressing cells on the confocal image (Fig. 4d and Additional file 1: Figure S3A). These results suggest that intracellular Chi3L1 is co-localized with p53 in the lung cancer cells and regulates the expression of p53.

### Intracellular Chi3L1 inhibits the transcriptional activity of p53 via the direct interaction in the lung cancer cells

**Fig. 5:**
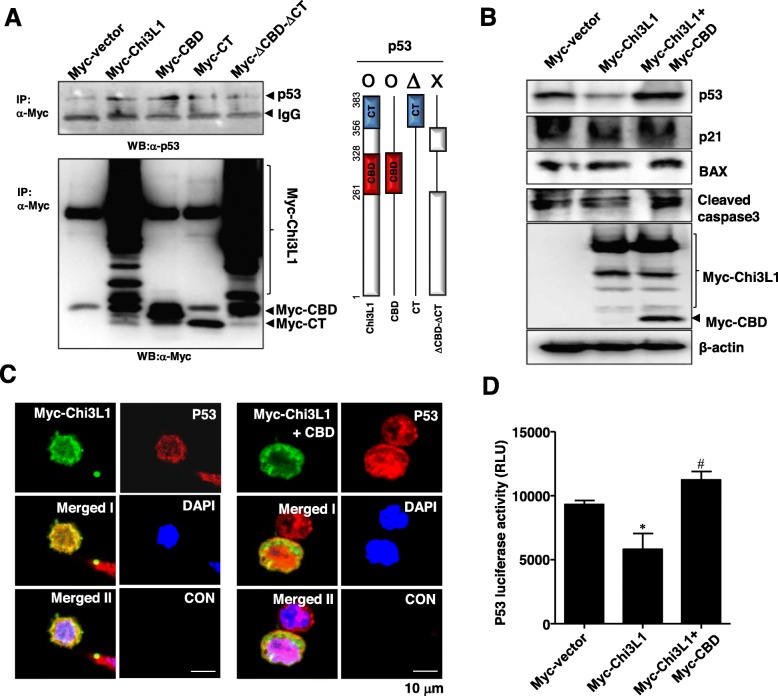
Effect of the CBD region of Chi3L1 on binding to p53 and its transcriptional activity (**a**) After the lung cancer cells were transfected with myc-vector, myc-Chi3L1, myc-CBD, myc-CT, and myc-ΔCBD-ΔCT, the cells were immunoprecipitated with anti-Myc antibody and then the immune complexes were then analyzed by Western blotting with anti-p53 antibody (*left*). Characterization of the structures in Chi3L1 that bind to p53 is illustrated (*right*). CBD: chitin binding domain, CT: C-terminal fragment. **b** The lung cancer cells were transfected with myc-vector, myc-Chi3L1, and myc-Chi3L1 + CBD. After then, the expression of myc-Chi3L1, p53, p21, BAX, cleaved caspase 3, and β-actin were analyzed by Western blotting. **c** The cells were fixed, permeabilized, and were analyzed by confocal microscopy for detection of intracellular co-localization for myc-Chi3L1 (green) and p53 (red). **d** p53 luciferase reporter vector that contains two copies of a p53 response element (p53 RE) were co-transfected with myc-vector, myc-Chi3L1, and myc-Chi3L1 + CBD for 24 h, the lysates were harvested and analyzed for luciferase activity. Data shown represent mean ± SEM. *, *p* < 0.05 compared to myc-vector. #, *p* < 0.05 compared to myc-Chi3L1

Deletion mapping was next employed to define the regions in Chi3L1 that are required for binding to p53 using co-IP assay. These studies demonstrated that the chitin binding domain (CBD) region of Chi3L1 between amino acids 261 and 328 was required to interact to p53, which is consistent with an interaction between Chi3L1 and p53 via molecular docking model (Fig. 5a, *third lane*). The c-terminal (CT) region of Chi3L1 between amino acids 356 and 383 did not play a critical role in this interaction (Fig. 5a, *fourth lane*). Deleted Chi3L1 of both CBD and CT regions did not interact with p53 (Fig. 5a, *fifth lane*). These results demonstrate that the physical interaction between Chi3L1 and p53 is mainly dependent on the CBD of Chi3L1.

To examine whether the downregulation of p53 by Chi3L1 in the lung cancer cells is mediated through the direct interaction between Chi3L1 and p53, we tried to interfere in this interaction by overexpression of the CBD region. As shown in Fig. 5b and Additional file 1: Figure S4A, the overexpression of the CBD region significantly abolished a decrease in the expression of p53 by myc-Chi3L1, which consequently attenuated effects of myc-Chi3L1 on expression of p21, BAX, and cleaved caspase 3 (Fig. 5b, *third lane*). We also confirmed the reduced expression of p53 mediated through the direct interaction using confocal microscopy (Fig. 5c and Additional file 1: Figure S5A). Then, we further investigated functional interaction where is based on physical interaction between Chi3L1 and p53 using p53 luciferase reporter assay. We demonstrated that Chi3L1 inhibits the transcriptional activity of p53, and this response was attenuated by the interference of the CBD region (Fig. 5d).

### Expression pattern of Chi3L1 is reversely correlated with expression pattern of p53, and intracellular Chi3L1 highly interacts with p53 in human lung cancer patients

**Fig. 6:**
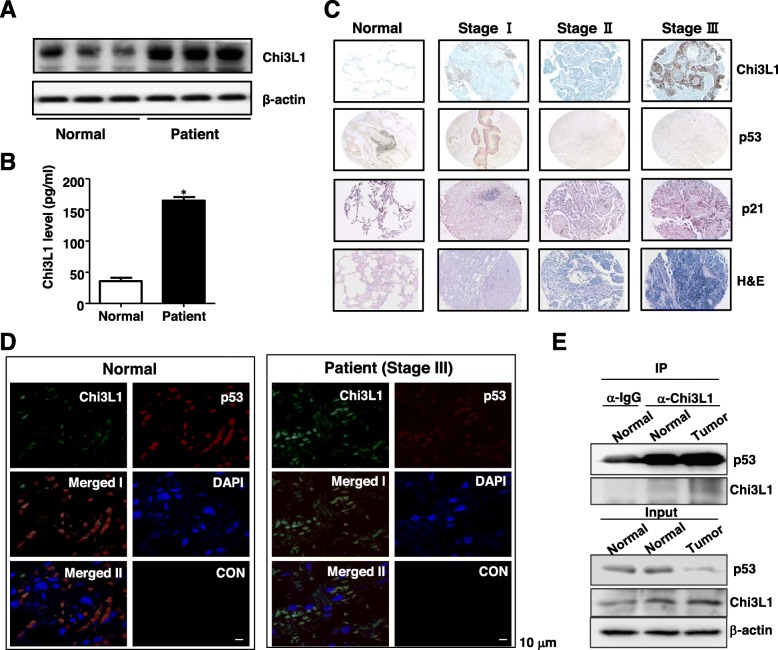
Human lung cancer patients in reverse expression, intracellular colocalization, and physical interaction between Chi3L1 and p53 in (**a**) Normal and lung tumor extracts which isolated from lung cancer patients were analyzed by Western blotting and detected using antibodies against Chi3L1 and β-actin. **b** The Chi3L1 level were detected by ELISA assay in lung cancer patients and normal person serum. **c** Human normal lung or tumor sections (Stage I–III) were processed and stained with Hematoxylin or analyzed by immunohistochemistry for detection of positive cells for Chi3L1, p53, p21, and p-STAT3. **d** After human patient tissues and normal lung tissues were permeabilized, the tissues were analyzed by confocal microscopy for detection of intracellular co-localization for Chi3L1 (green) and p53 (red). And then sections were stained with DAPI (*blue*). Merged I shows the merged images of Chi3L1 and p53, and Merged II shows the merged images of Chi3L1, p53, and DAPI. Negative control experiments (CON) were processed with only Alex568- and Alex488-conjugated IgG antibodies. **e** Human patient tissues and normal lung tissues were lysed and immunoprecipitated with anti-control IgG or anti-Chi3L1 antibodies. The immunocomplexes (*IP*) and extracts (*Input*) were then analyzed by Western blotting with anti-Chi3L1 and anti-p53 antibodies

To clear the expression of Chi3L1 on the lung tumor development in human, we performed Western blot assay using tumor tissues of human lung cancer patient. This study demonstrated that the expression of Chi3L1 was significantly increased in tumor tissues of human lung cancer patient compared to human normal lung tissues (Fig. 6a). We also found that the level of Chi3L1 significantly was elevated in serum of human lung cancer patient compared to normal serum (Fig. 6b). To further determine the pathological relevance between intracellular Chi3L1 and p53 expression in lung cancer patients, we examined whether the expression of Chi3L1 was related with p53 expression in different stage of lung tumor patients tissues using application of human tissue microarray. In immunohistochemical staining, Chi3L1 expression was strongly increased in human lung tumor tissues at a stage dependent manner. We also investigated the expression of p53, a target protein of Chi3L1. The expression pattern of p53 was lower in tumor tissues compared to that in normal tissues (Fig. 6c). Thus, these data support the reliability that intracellular Chi3L1 and p53 play a role in the human lung cancer development.

To order to validate the intracellular relationship between Chi3L1 and p53, we performed Immunofluorescence analysis in tissues of human lung cancer patient. As shown in Fig. 6d, the expression of Chi3L1 was increased in tissues of human lung cancer patient compared to human normal lung tissues, whereas the expression of p53 was decreased in human lung cancer patient compared to human normal lung tissues. Moreover, Immunofluorescence analysis indicated intracellular co-localization between Chi3L1 and p53 in human tissues (Fig. 6d). Accompanied with co-localization, we demonstrated that physical interaction of intracellular Chi3L1 with p53 was increased in tissues of lung cancer patient (Fig. 6e). Our data suggest that intracellular Chi3L1 suppressed the expression and function of p53 in human lung cancer patients.

## Discussion

Chi3L1 do not possess chitinase activity and fails to degrade chitin due to amino acid mutations within the proposed catalytic site (conserved sequence: DXXDXDXE; YKL-40 sequence: DGLDLAWL) although Chi3L1 has homologous sequence to bacterial and fungal chitinases [11, 38]. High expression of Chi3L1 is found in pathological conditions [20–31], but the clinical implications and function of Chi3L1 are still elusive. In these experiments, we generated Chi3L1^KO(−/−)^ mice using CRISPR/Cas9 system and found that the Chi3L1^KO(−/−)^ mice significantly reduced lung tumorigenesis. This effect was coincident with the increased p53 expression which led to cell cycle arrest and apoptotic signaling. Lower tumor growth by the downregulation of shRNA-induced Chi3L1 was also associated with increased p53 expression with its target proteins in in vivo allograft mice. Moreover, Chi3L1 knockdown in lung cancer cell significantly inhibited lung cancer cell growth accompanied with increased p53 expression and its target proteins. These data indicate that the expression of Chi3L1 promotes lung tumorigenesis via decreasing tumor suppressor proteins, followed by an increase in cell cycle arrest and apoptosis, but a decrease in proliferation.

Recent studies demonstrated that the secreted Chi3L1 directly binds to IL-13 receptor α2 (IL-13Rα2) that was originally described a high affinity receptor for IL-13 and believed to be a decoy receptor for IL-13 [39–42]. The extracellular interaction between IL-13Rα2 and Chi3L1 regulates pathogen responses, oxidant injury, inflammation, and melanoma metastasis [39], and also Chi3L1 activates the Wnt/β-catenin, mitogen-activated protein kinase (MAPK), and Protein kinase B (PKB/AKT) signaling pathways via IL-13Rα2 [39] In addition, transmembrane protein 219 (TMEM219), a membrane protein plays a critical role in Chi3L1-induced IL-13Rα2 mediated signaling and responses including oxidant-induced apoptosis, lung injury, and melanoma metastasis [40]. When viewed in combination and based on the previous literatures [39–41, 43–46], it is supposed that Chi3L1 is secreted and extracellular Chi3L1 regulates lung metastasis and pathological phenomenon via putative membrane proteins. However, it is still unclear how Chi3L1 accomplishes these varied responses and how Chi3L1 induces lung tumorigenesis.

Interestingly, it is reported that Chi3L1 was localized within the cytoplasm and nucleus in monocyte derived dendritic cells, and Chi3L1 even possesses nuclear localization sequence (RRDKQHFTTLIKEMKAE-FIKEAQPGKKQLL) that could be localized in the nucleus and in the cytoplasm [32], suggesting that the increase of intracellular Chi3L1 during the differentiation and the maturation of dendritic cells may play a new role in the transcriptional process of dendritic cells [32]. In the present study, we also found that Chi3L1 is placed within the cytoplasm and nucleus in the lung cancer cells. According to the diseases and signaling network, p53 is closely related with Chi3L1 and our results demonstrated that p53 is significantly increased in Chi3L1^KO(−/−)^ mice. Similar with this data, p53 was also significantly increased in downregulation of Chi3L1 in allograft mice and lung cancer cells. Chi3L1 also suppressed p53 transcriptional activity via the direct interaction mediated by CBD region of Chi3L1. Concomitantly, the expression of p21 and BAX was increased under the same condition. These results are in agreement with previous reports for p53, a well known transcriptional factor that functions as a tumor suppressor [47, 48]. p53 is activated in response to various oncogenic stresses and, thus, plays crucial roles in cancer prevention of tumor formation through cell cycle arrest, apoptosis, or autophagy [47–49]. Therefore, our data suggest that p53 is a novel binding partner of intracellular Chi3L1 and the physical interaction is essential in lung tumorigenesis by inhibiting tumor suppresser proteins.

p53 is targeted for ubiquitination and degradation by directly various E3 ubiquitin ligases, including MDM2 and Parkin [49, 50]. In the present study, we demonstrated that the interaction of Chi3L1 with p53 induces p53 ubiquitination. Molecular docking model of Chi3L1 with p53 and also the deletion mutants of Chi3L1 revealed the direct interactions with p53 in CBD region of Chi3L1. Since Jab1 indirectly enhances MDM2-mediated p53 ubiquitination [51, 52] and also YY1 (Yin Yang 1) binds to MDM2 and indirectly enhances p53 ubiquitination and degradation [53], our data suggest that Chi3L1 negatively regulates p53 by inducing ubiquitination even if Chi3L1 is not a E3 ubiquitin ligase. On the other hand, it was reported that the increased levels of Chi3L1 occur in inhibition of an effective p53 signaling in U87 cells by siRNA or cyclic pifithrin-α, and the expression of Chi3L1 was elevated both on RNA levels and protein levels [54], suggesting that p53 stabilization exerts an inhibitory influence on Chi3L1 production although the study was not investigated in mutant p53 protein. Considering this paper and our data, one could speculate that the activity and expression of p53 were negatively modulated by Chi3L1 and vice versa. To be more accurate, Chi3L1 affects p53 stabilization and its transcriptional activities on protein levels, while p53 transcriptionally affects Chi3L1 expression on mRNA level, indicating that the balance between p53 and Chi3L1 is strictly regulated under pathophysiologcal condtions. In human lung tumor patients, we also validated in vitro and in vivo experiments. The intracellular colocalization and interaction between Chi3L1 and p53 occurred, as well as the expression pattern of Chi3L1 also showed reverse correlation with the expression pattern of p53 in human lung tumor patients.

## Conclusion

Our finding is the first report demonstrating the critical role and mechanisms of intracellular Chi3L1-mediated p53 inhibition through direct interaction in the lung tumorigenesis. Thus, the crucial roles identified for intracellular Chi3L1 in a new context provide new insight on molecular and clinical pathology into lung tumorigenesis, and suggest that Chi3L1 may be a potential target for the development of new therapeutic agents.

## Supplementary information

**Additional file 1 **: **Figure S1.** Regulation of Chi3L1 on lung cancer cell growth. (A) After A549 lung cancer cells were transfected with Chi3L1 siRNA for 24 h, the morphological changes were observed, and then cell proliferation was analyzed by BrdU incorporation assay. Data shown represent mean ± SEM. *, *p* < 0.05 compared to con siRNA. **Figure S2.** Bar graph for western blot bands in Fig. 3c (A - F) The bar graph represents densitometry data from Chi3L1 (A), p53 (B), p21 (C), BAX (D), cleaved caspase 3 (E), and phoshp-STAT3 (F). Relative level (%) was normalized to β-actin and relatively quantified to the amount in cells transfected with con siRNA. Data shown represent mean ± SEM. *, *p* < 0.05 compared to con siRNA. **Figure S3.** Bar graph for confocal images in Fig. 4d (A) The bar graph represents densitometry data from p53 (red). Relative level (fold) was normalized to the amount of p53 in Myc-Chi3L1-non-expressing cell. Arrow: Myc-Chi3L1-expressing cell, Arrow head: Myc-Chi3L1-non-expressing cell. **Figure S4.** Bar graph for western blot bands in Fig. 5b (A) The bar graph represents densitometry data from p53. Relative level (%) was normalized to β-actin and relatively quantified to the amount in cells transfected with Myc-Vector. Data shown represent mean ± SEM. *, *p* < 0.05 compared to Myc-Vector. **Figure S5.** Bar graph for confocal images in Fig. 5c (A) The bar graph represents densitometry data from p53 (red). Relative level (fold) was normalized to the amount of p53 in Myc-Chi3L1-expressing cell transfected with Myc-Chi3L1.

## Data Availability

Not applicable.

## References

[1] Takahashi T (2002). Lung cancer: an ever increasing store of in-depth basic knowledge and the beginning of its clinical application. Oncogene.

[2] Thun MJ, Hannan LM, Adams-Campbell LL, Boffetta P, Buring JE, Feskanich D, Flanders WD, Jee SH, Katanoda K, Kolonel LN (2008). Lung cancer occurrence in never-smokers: an analysis of 13 cohorts and 22 cancer registry studies. PLoS Med.

[3] Scarpace SL (2015). Metastatic squamous cell non-small-cell lung cancer (NSCLC): disrupting the drug treatment paradigm with immunotherapies. Drugs Context.

[4] Scheff RJ, Schneider BJ (2013). Non-small-cell lung cancer: treatment of late stage disease: chemotherapeutics and new frontiers. Semin Intervent Radiol.

[5] Mendez M, Custodio A, Provencio M (2011). New molecular targeted therapies for advanced non-small-cell lung cancer. J Thorac Dis.

[6] Son DJ, Zheng J, Jung YY, Hwang CJ, Lee HP, Woo JR, Baek SY, Ham YW, Kang MW, Shong M (2017). MMPP attenuates non-small cell lung Cancer growth by inhibiting the STAT3 DNA-binding activity via direct binding to the STAT3 DNA-binding domain. Theranostics.

[7] Coudert B, Anthoney A, Fiedler W, Droz JP, Dieras V, Borner M, Smyth JF, Morant R, de Vries MJ, Roelvink M, Fumoleau P (2001). Phase II trial with ISIS 5132 in patients with small-cell (SCLC) and non-small cell (NSCLC) lung cancer. A European Organization for Research and Treatment of Cancer (EORTC) early clinical studies group report. Eur J Cancer.

[8] Soria JC, Shepherd FA, Douillard JY, Wolf J, Giaccone G, Crino L, Cappuzzo F, Sharma S, Gross SH, Dimitrijevic S (2009). Efficacy of everolimus (RAD001) in patients with advanced NSCLC previously treated with chemotherapy alone or with chemotherapy and EGFR inhibitors. Ann Oncol.

[9] Zarogoulidis P, Chinelis P, Athanasiadou A, Tsiouda T, Trakada G, Kallianos A, Veletza L, Hatzibougias D, Mihalopoulou E, Goupou E (2017). Possible adverse effects of immunotherapy in non-small cell lung cancer; treatment and follow-up of three cases. Respir Med Case Rep.

[10] Ruiz-Banobre J, Perez-Pampin E, Garcia-Gonzalez J, Gomez-Caamano A, Baron-Duarte FJ, Lopez-Lopez R, Vazquez-Rivera F (2017). Development of psoriatic arthritis during nivolumab therapy for metastatic non-small cell lung cancer, clinical outcome analysis and review of the literature. Lung Cancer.

[11] Hakala BE, White C, Recklies AD (1993). Human cartilage gp-39, a major secretory product of articular chondrocytes and synovial cells, is a mammalian member of a chitinase protein family. J Biol Chem.

[12] Rejman JJ, Hurley WL (1988). Isolation and characterization of a novel 39 kilodalton whey protein from bovine mammary secretions collected during the nonlactating period. Biochem Biophys Res Commun.

[13] Harvey S, Weisman M, O'Dell J, Scott T, Krusemeier M, Visor J, Swindlehurst C (1998). Chondrex: new marker of joint disease. Clin Chem.

[14] Kamba A, Lee IA, Mizoguchi E (2013). Potential association between TLR4 and chitinase 3-like 1 (CHI3L1/YKL-40) signaling on colonic epithelial cells in inflammatory bowel disease and colitis-associated cancer. Curr Mol Med.

[15] Yang MS, Morris DW, Donohoe G, Kenny E, O'Dushalaine CT, Schwaiger S, Nangle JM, Clarke S, Scully P, Quinn J (2008). Chitinase-3-like 1 (CHI3L1) gene and schizophrenia: genetic association and a potential functional mechanism. Biol Psychiatry.

[16] Kang MJ, Yoon CM, Nam M, Kim DH, Choi JM, Lee CG, Elias JA (2015). Role of Chitinase 3-Like-1 in Interleukin-18-induced pulmonary type 1, type 2, and type 17 inflammation; alveolar destruction; and airway fibrosis in the murine lung. Am J Respir Cell Mol Biol.

[17] Abe K, Nakamura Y, Yamauchi K, Maemondo M (2018). Role of genetic variations of chitinase 3-like 1 in bronchial asthmatic patients. Clin Mol Allergy.

[18] Di Rosa M, Malaguarnera L (2016). Chitinase 3 Like-1: an emerging molecule involved in diabetes and diabetic complications. Pathobiology.

[19] Jung YY, Kim KC, Park MH, Seo Y, Park H, Chang J, Hwang DY, Han SB, Kim S, Son DJ, Hong JT (2018). Atherosclerosis is exacerbated by chitinase-3-like-1 in amyloid precursor protein transgenic mice. Theranostics.

[20] Libreros S, Garcia-Areas R, Iragavarapu-Charyulu V (2013). CHI3L1 plays a role in cancer through enhanced production of pro-inflammatory/pro-tumorigenic and angiogenic factors. Immunol Res.

[21] Ngernyuang N, Shao R, Suwannarurk K, Limpaiboon T (2018). Chitinase 3 like 1 (CHI3L1) promotes vasculogenic mimicry formation in cervical cancer. Pathology.

[22] Kim DH, Park HJ, Lim S, Koo JH, Lee HG, Choi JO, Oh JH, Ha SJ, Kang MJ, Lee CM (2018). Regulation of chitinase-3-like-1 in T cell elicits Th1 and cytotoxic responses to inhibit lung metastasis. Nat Commun.

[23] Xing S, Zheng X, Zeng T, Zeng MS, Zhong Q, Cao YS, Pan KL, Wei C, Hou F, Liu WL (2017). Chitinase 3-like 1 secreted by peritumoral macrophages in esophageal squamous cell carcinoma is a favorable prognostic factor for survival. World J Gastroenterol.

[24] Erturk K, Tas F, Serilmez M, Bilgin E, Yasasever V (2017). Clinical significance of serum Ykl-40 (Chitinase-3-Like-1 protein) as a biomarker in melanoma: an analysis of 112 Turkish patients. Asian Pac J Cancer Prev.

[25] Cohen N, Shani O, Raz Y, Sharon Y, Hoffman D, Abramovitz L, Erez N (2017). Fibroblasts drive an immunosuppressive and growth-promoting microenvironment in breast cancer via secretion of Chitinase 3-like 1. Oncogene.

[26] Huang WS, Lin HY, Yeh CB, Chen LY, Chou YE, Yang SF, Liu YF (2017). Correlation of Chitinase 3-like 1 single nucleotide polymorphisms with hepatocellular carcinoma in Taiwan. Int J Med Sci.

[27] Park MH, Yun HM, Hwang CJ, Park SI, Han SB, Hwang DY, Yoon DY, Kim S, Hong JT (2017). Presenilin mutation suppresses lung tumorigenesis via inhibition of Peroxiredoxin 6 activity and expression. Theranostics.

[28] Hogdall EV, Ringsholt M, Hogdall CK, Christensen IJ, Johansen JS, Kjaer SK, Blaakaer J, Ostenfeld-Moller L, Price PA, Christensen LH (2009). YKL-40 tissue expression and plasma levels in patients with ovarian cancer. BMC Cancer.

[29] Bernardi D, Padoan A, Ballin A, Sartori M, Manara R, Scienza R, Plebani M, Della Puppa A (2012). Serum YKL-40 following resection for cerebral glioblastoma. J Neuro-Oncol.

[30] Furuhashi K, Suda T, Nakamura Y, Inui N, Hashimoto D, Miwa S, Hayakawa H, Kusagaya H, Nakano Y, Nakamura H, Chida K (2010). Increased expression of YKL-40, a chitinase-like protein, in serum and lung of patients with idiopathic pulmonary fibrosis. Respir Med.

[31] Johansen JS, Drivsholm L, Price PA, Christensen IJ (2004). High serum YKL-40 level in patients with small cell lung cancer is related to early death. Lung Cancer.

[32] Di Rosa M, Tibullo D, Saccone S, Distefano G, Basile MS, Di Raimondo F, Malaguarnera L (2016). CHI3L1 nuclear localization in monocyte derived dendritic cells. Immunobiology.

[33] Choi IK, Kim YH, Kim JS, Seo JH (2010). High serum YKL-40 is a poor prognostic marker in patients with advanced non-small cell lung cancer. Acta Oncol.

[34] Jao LE, Wente SR, Chen W (2013). Efficient multiplex biallelic zebrafish genome editing using a CRISPR nuclease system. Proc Natl Acad Sci U S A.

[35] Ittner LM, Gotz J (2007). Pronuclear injection for the production of transgenic mice. Nat Protoc.

[36] Yun HM, Kim S, Kim HJ, Kostenis E, Kim JI, Seong JY, Baik JH, Rhim H (2007). The novel cellular mechanism of human 5-HT6 receptor through an interaction with Fyn. J Biol Chem.

[37] Park KR, Kim JY, Kim EC, Yun HM, Hong JT (2017). RANKL-induced osteoclastogenesis is suppressed by 4-O-methylhonokiol in bone marrow-derived macrophages. Arch Pharm Res.

[38] Renkema GH, Boot RG, Au FL, Donker-Koopman WE, Strijland A, Muijsers AO, Hrebicek M, Aerts JM (1998). Chitotriosidase, a chitinase, and the 39-kDa human cartilage glycoprotein, a chitin-binding lectin, are homologues of family 18 glycosyl hydrolases secreted by human macrophages. Eur J Biochem.

[39] He CH, Lee CG, Dela Cruz CS, Lee CM, Zhou Y, Ahangari F, Ma B, Herzog EL, Rosenberg SA, Li Y (2013). Chitinase 3-like 1 regulates cellular and tissue responses via IL-13 receptor alpha2. Cell Rep.

[40] Lee CM, He CH, Nour AM, Zhou Y, Ma B, Park JW, Kim KH, Dela Cruz C, Sharma L, Nasr ML (2016). IL-13Ralpha2 uses TMEM219 in chitinase 3-like-1-induced signalling and effector responses. Nat Commun.

[41] Zhou Y, He CH, Yang DS, Nguyen T, Cao Y, Kamle S, Lee CM, Gochuico BR, Gahl WA, Shea BS (2018). Galectin-3 interacts with the CHI3L1 Axis and contributes to Hermansky-Pudlak syndrome lung disease. J Immunol.

[42] Fichtner-Feigl S, Strober W, Kawakami K, Puri RK, Kitani A (2006). IL-13 signaling through the IL-13alpha2 receptor is involved in induction of TGF-beta1 production and fibrosis. Nat Med.

[43] Kawada M, Chen CC, Arihiro A, Nagatani K, Watanabe T, Mizoguchi E (2008). Chitinase 3-like-1 enhances bacterial adhesion to colonic epithelial cells through the interaction with bacterial chitin-binding protein. Lab Investig.

[44] Low D, Tran HT, Lee IA, Dreux N, Kamba A, Reinecker HC, Darfeuille-Michaud A, Barnich N, Mizoguchi E (2013). Chitin-binding domains of Escherichia coli ChiA mediate interactions with intestinal epithelial cells in mice with colitis. Gastroenterology.

[45] Chen CC, Llado V, Eurich K, Tran HT, Mizoguchi E (2011). Carbohydrate-binding motif in chitinase 3-like 1 (CHI3L1/YKL-40) specifically activates Akt signaling pathway in colonic epithelial cells. Clin Immunol.

[46] Lee IA, Low D, Kamba A, Llado V, Mizoguchi E (2014). Oral caffeine administration ameliorates acute colitis by suppressing chitinase 3-like 1 expression in intestinal epithelial cells. J Gastroenterol.

[47] Vousden KH, Prives C (2009). Blinded by the light: the growing complexity of p53. Cell.

[48] Menendez D, Inga A, Resnick MA (2009). The expanding universe of p53 targets. Nat Rev Cancer.

[49] Inoue K, Fry EA, Frazier DP (2016). Transcription factors that interact with p53 and Mdm2. Int J Cancer.

[50] Jung YY, Son DJ, Lee HL, Kim DH, Song MJ, Ham YW, Kim Y, Han SB, Park MH, Hong JT (2017). Loss of Parkin reduces inflammatory arthritis by inhibiting p53 degradation. Redox Biol.

[51] Lee EW, Oh W, Song J (2006). Jab1 as a mediator of nuclear export and cytoplasmic degradation of p53. Mol Cells.

[52] Oh W, Lee EW, Sung YH, Yang MR, Ghim J, Lee HW, Song J (2006). Jab1 induces the cytoplasmic localization and degradation of p53 in coordination with Hdm2. J Biol Chem.

[53] Sui G, el Affar B, Shi Y, Brignone C, Wall NR, Yin P, Donohoe M, Luke MP, Calvo D, Grossman SR (2004). Yin Yang 1 is a negative regulator of p53. Cell.

[54] Junker N, Johansen JS, Hansen LT, Lund EL, Kristjansen PE (2005). Regulation of YKL-40 expression during genotoxic or microenvironmental stress in human glioblastoma cells. Cancer Sci.

