# Supplementary figures and images for: Chitinase 3 like 1 suppresses the stability and activity of p53 to promote lung tumorigenesis
Source: Cell Commun Signal. 2020 Mar 4;18:5. doi: 10.1186/s12964-019-0503-7 (PMC7055043; doi:10.1186/s12964-019-0503-7)

**A**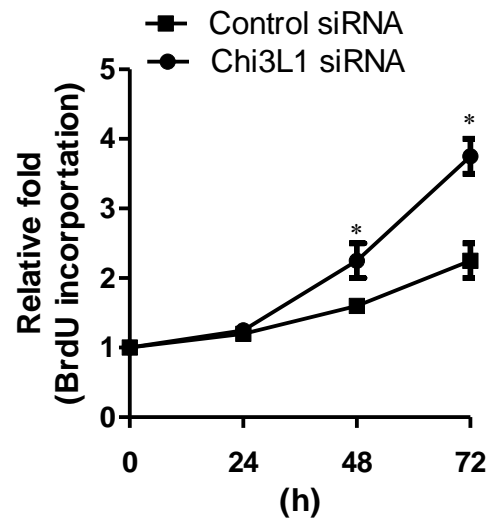

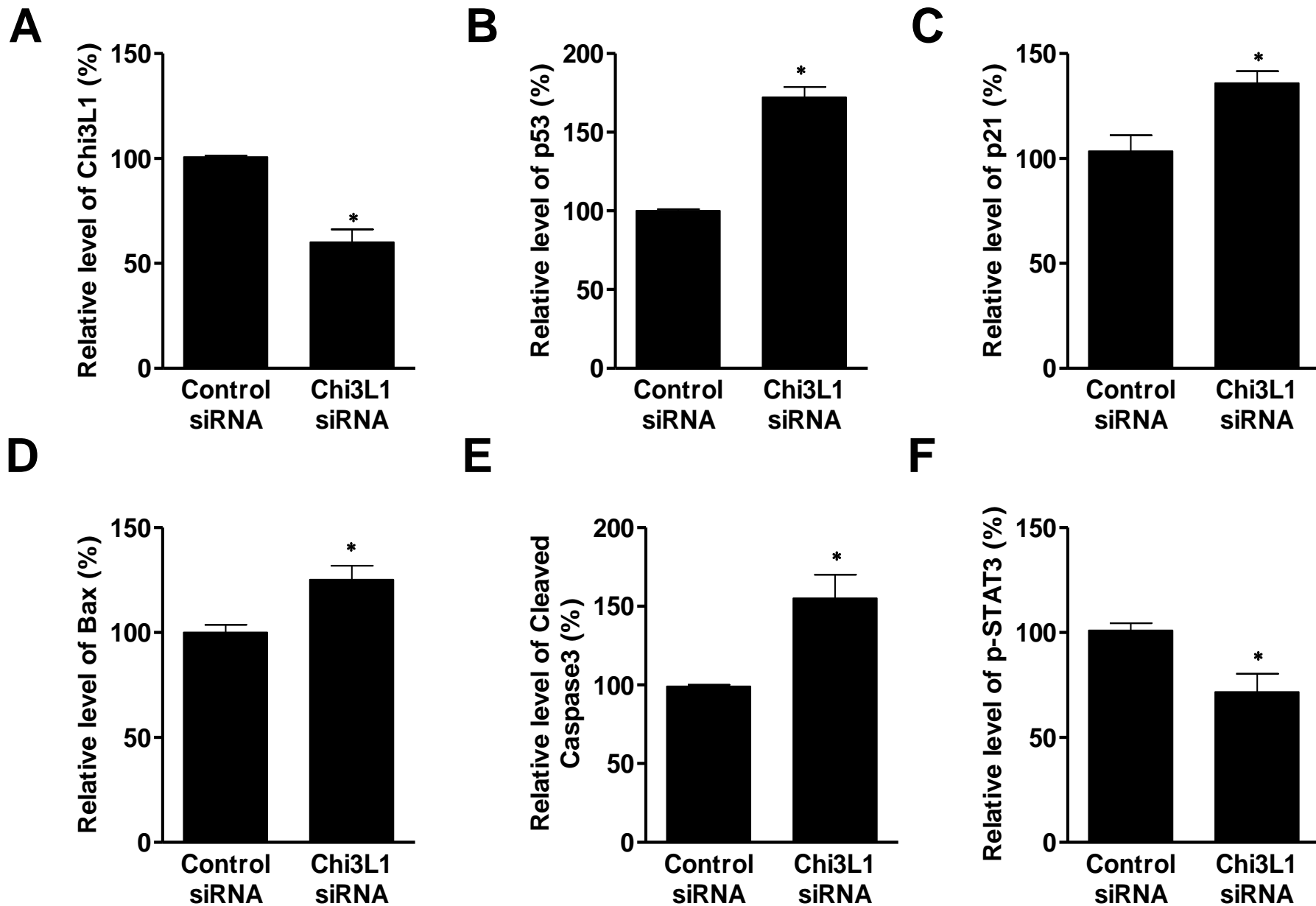

**A**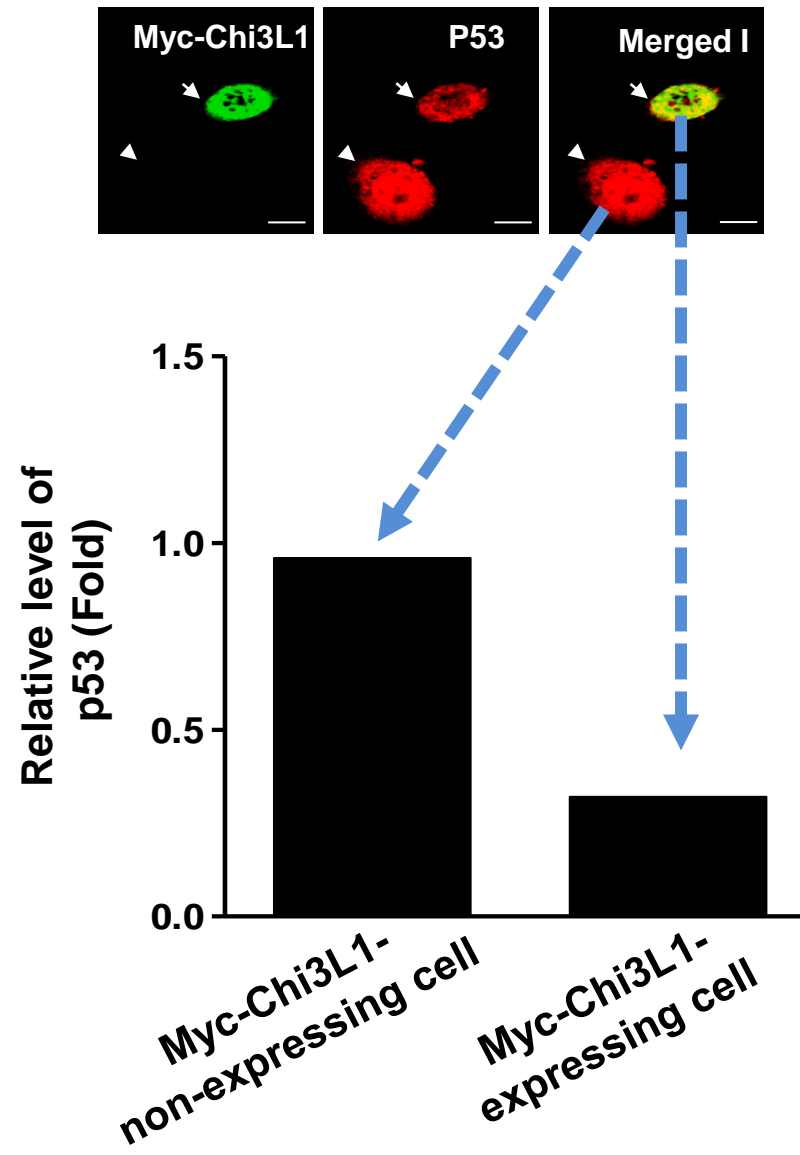

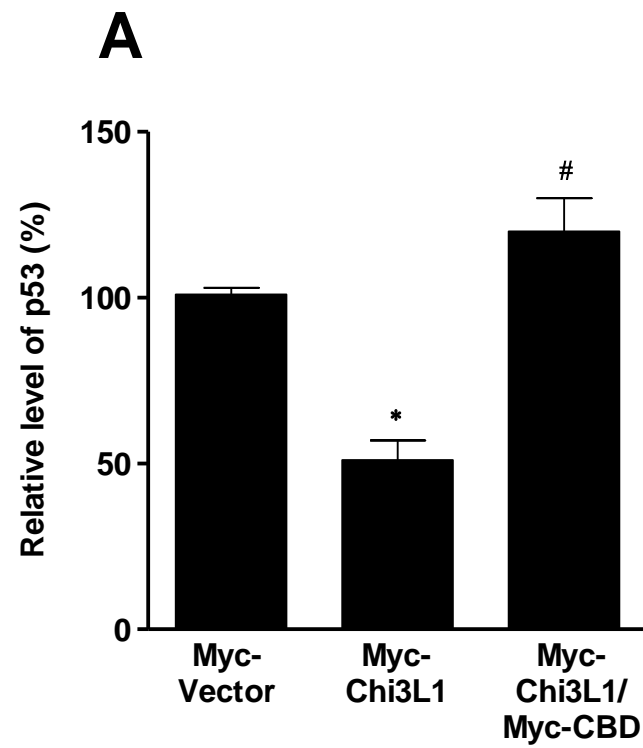

A

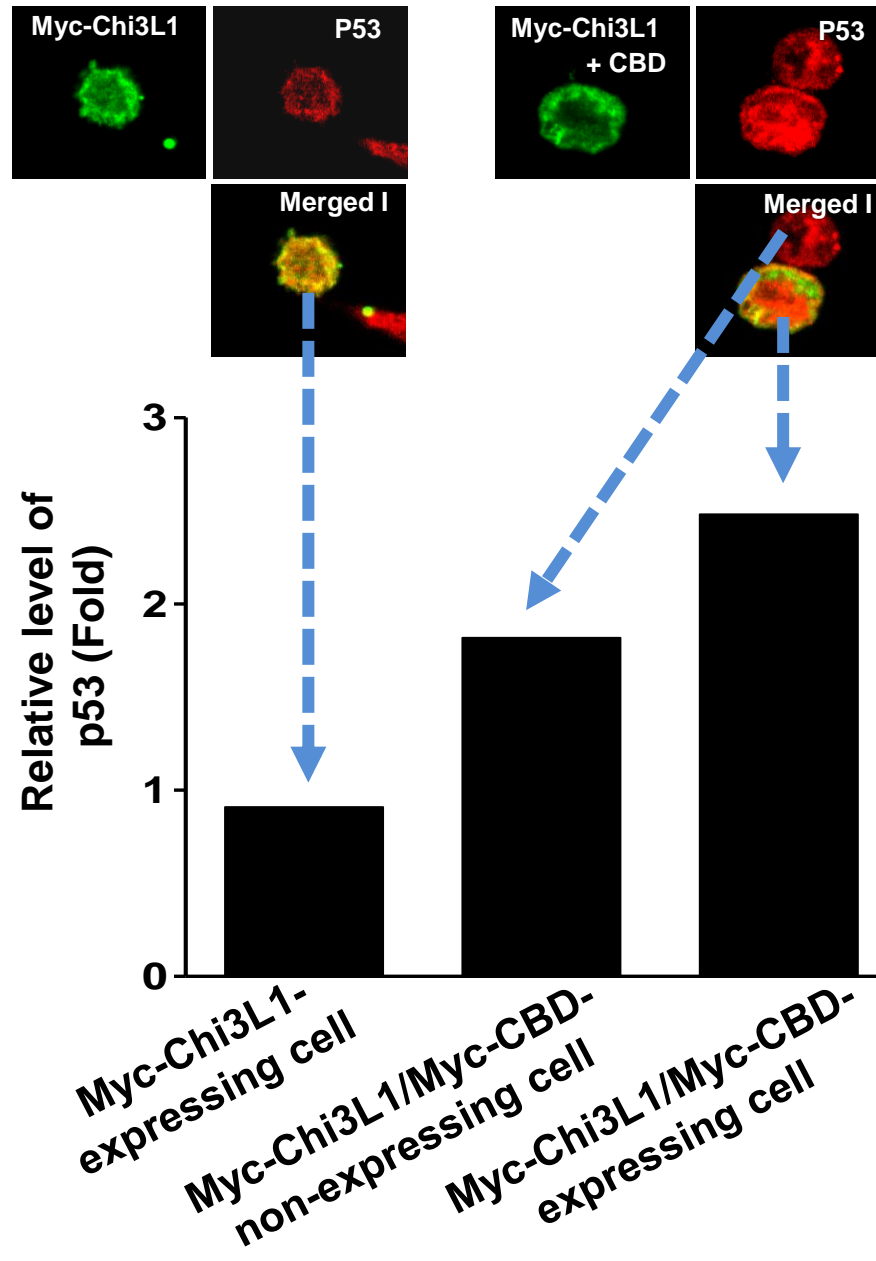

Supplement: Supplementary file 1 — Additional file 1 : Figure S1. Regulation of Chi3L1 on lung cancer cell growth. (A) After A549 lung cancer cells were transfected with Chi3L1 siRNA for 24 h, the morphological changes were observed, and then cell proliferation was analyzed by BrdU incorporation assay. Data shown represent mean ± SEM. *, p < 0.05 compared to con siRNA. Figure S2. Bar graph for western blot bands in Fig. 3c (A - F) The bar graph represents densitometry data from Chi3L1 (A), p53 (B), p21 (C), BAX (D), cleaved caspase 3 (E), and phoshp-STAT3 (F). Relative level (%) was normalized to β-actin and relatively quantified to the amount in cells transfected with con siRNA. Data shown represent mean ± SEM. *, p < 0.05 compared to con siRNA. Figure S3. Bar graph for confocal images in Fig. 4d (A) The bar graph represents densitometry data from p53 (red). Relative level (fold) was normalized to the amount of p53 in Myc-Chi3L1-non-expressing cell. Arrow: Myc-Chi3L1-expressing cell, Arrow head: Myc-Chi3L1-non-expressing cell. Figure S4. Bar graph for western blot bands in Fig. 5b (A) The bar graph represents densitometry data from p53. Relative level (%) was normalized to β-actin and relatively quantified to the amount in cells transfected with Myc-Vector. Data shown represent mean ± SEM. *, p < 0.05 compared to Myc-Vector. Figure S5. Bar graph for confocal images in Fig. 5c (A) The bar graph represents densitometry data from p53 (red). Relative level (fold) was normalized to the amount of p53 in Myc-Chi3L1-expressing cell transfected with Myc-Chi3L1. [file 12964_2019_503_MOESM1_ESM.pdf]
